# Voxelisation Algorithms and Data Structures: A Review

**DOI:** 10.3390/s21248241

**Published:** 2021-12-09

**Authors:** Mitko Aleksandrov, Sisi Zlatanova, David J. Heslop

**Affiliations:** 1 The School of Built Environment, The University of New South Wales, Sydney, NSW 2052, Australia; s.zlatanova@unsw.edu.au; 2 The School of Public Health and Community Medicine, The University of New South Wales, Sydney, NSW 2052, Australia; d.heslop@unsw.edu.au

**Keywords:** voxel, voxelisation, data structures, algorithms, geometric primitives

## Abstract

Voxel-based data structures, algorithms, frameworks, and interfaces have been used in computer graphics and many other applications for decades. There is a general necessity to seek adequate digital representations, such as voxels, that would secure unified data structures, multi-resolution options, robust validation procedures and flexible algorithms for different 3D tasks. In this review, we evaluate the most common properties and algorithms for voxelisation of 2D and 3D objects. Thus, many voxelisation algorithms and their characteristics are presented targeting points, lines, triangles, surfaces and solids as geometric primitives. For lines, we identify three groups of algorithms, where the first two achieve different voxelisation connectivity, while the third one presents voxelisation of curves. We can say that surface voxelisation is a more desired voxelisation type compared to solid voxelisation, as it can be achieved faster and requires less memory if voxels are stored in a sparse way. At the same time, we evaluate in the paper the available voxel data structures. We split all data structures into static and dynamic grids considering the frequency to update a data structure. Static grids are dominated by SVO-based data structures focusing on memory footprint reduction and attributes preservation, where SVDAG and SSVDAG are the most advanced methods. The state-of-the-art dynamic voxel data structure is NanoVDB which is superior to the rest in terms of speed as well as support for out-of-core processing and data management, which is the key to handling large dynamically changing scenes. Overall, we can say that this is the first review evaluating the available voxelisation algorithms for different geometric primitives as well as voxel data structures.

## 1. Introduction

Voxel-based representations are used in many application domains. In computer graphics, voxels are used for fast ray tracing [1], voxelisation of surfaces and solids [2,3], shadow [4] and visibility analysis [5,6,7]. These are mostly focused on fast-real time visualisation and therefore aiming at visualising only visible voxels. In medicine, voxel representations are commonly implemented in software processing CT and MRI scans investigating organs and body structure in three dimensions [8,9]. Voxel approaches are increasingly being used in city modelling for 3D reconstruction [10] and spatial analysis [11]. Voxel-based models are commonly investigated with the aim to define geological phenomena [12,13]. In environmental analysis, voxelisation is used to establish computational domains for gaseous and liquid simulations as well as to interact with obstacles [14]. Voxel-based methods are extensively applied for the processing of point clouds [15,16,17]. A quick voxelisation is suggested to derive navigable areas for pedestrian simulation [18,19,20] and collision detection [21,22]. As we can see voxels are highly applicable in various domains due to their discrete representation creating a continuous phenomenon in space.

A discrete approximation of digital objects or continuous phenomena is called voxelisation. Many different voxelisations are performed targeting lines [23], triangles [24], polygons [25], surfaces [26] and solids [2,3]. When voxelising 3D objects different properties can be considered such as connectivity, separation, coverage and tunnelling [27], as well as colours preservation [28] and anti-aliasing [29] which are related to non-binary voxelisation. Based on our knowledge there is no single paper that evaluates and presents the characteristics of available algorithms for the voxelisation of different geometrical primitives.

Although dense regular grids are convenient to use for several reasons, their main disadvantage is their memory footprint which is directly proportional to the volume of embedding space. Since areas including 3D city modelling and physical simulations usually deal with data that occupy only a fraction of the entire space, sparse representation of volumetric data is suggested keeping only voxels that contain meaningful information [3,30]. Apart from data structures such as sparse octrees that tend to reduce the memory needs for rendering large objects and scenes, numerous 3D sparse data structures are more adaptive suiting the needs of simulations [31,32]. However, to be able to scale to extreme resolutions and handle physical simulations with dynamic topology more balanced and generic data structures are developed [33,34]. Thus, identifying the characteristics of available 3D data volumetric structures is required to understand their pros and cons for different applications.

To be able to work with voxels, the conversion from vector-based primitives such as points, lines, triangles, surfaces and solids needs to be performed. This paper presents a review on methods for voxelisation and voxel data structures, dealing with geometry, properties and semantics of objects. The paper is organised as follows. Section 2 introduces the main properties and principles related to discrete space that guide a voxelisation process as well as binary and non-binary voxelisation. Section 3 explores algorithms for the voxelisation of different 3D geometric primitives. Section 4 discusses various aspects related to voxel hardware technology and data structures available today to effectively manage voxels for different applications. Section 5 highlights some final remarks and conclusions based on the entire review.

## 2. Voxelisation Properties

The literature provides many definitions of a voxel space. Here we will refer to a voxels space as to an integer space. Let Z^3^ be the subset of 3D Euclidian space R^3^ that is represented by all points whose coordinates are integers. This subset is called a *grid* [27]. A grid point represents a cell commonly referred to as a voxel. Voxels can have multiple properties, which can be organised differently with respect to the application. Binary voxelisation is a term to indicate that a voxel can have a property, which can take only two values: 0 (empty) or 1 (filled).

**Figure 1:**
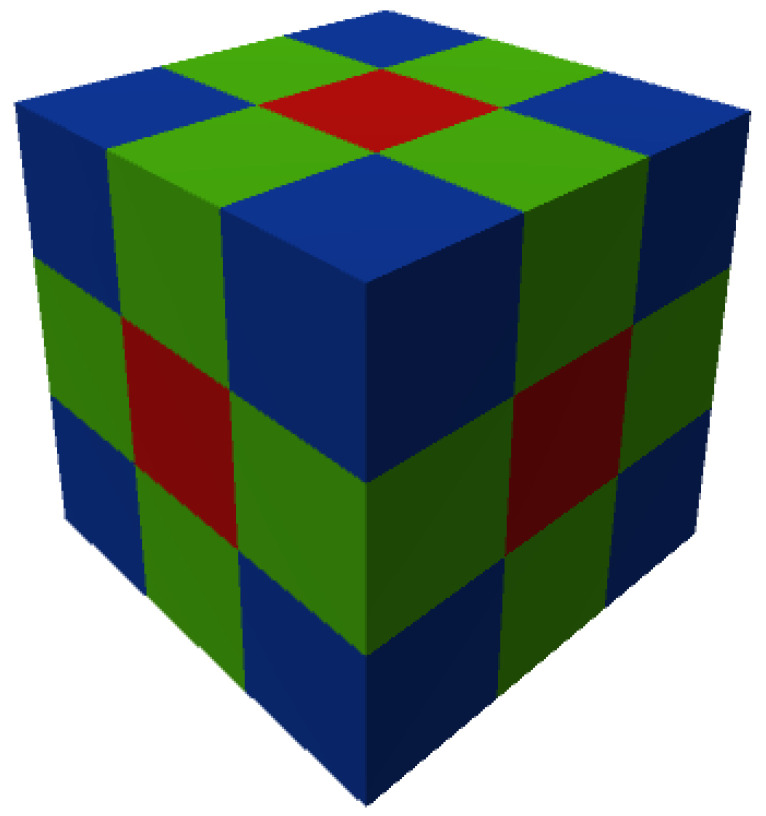
The 26 neighbours of a voxel; six voxels sharing face (in red), 12 voxels sharing edge (in green) and 8 voxels sharing a vertex (in blue).

The shape of a voxel is generally considered as a cube, although applications may use cuboid representations [13]. The neighbourhood properties of a voxel play an important role in all voxel-based algorithms. A voxel can have a maximum of 26 adjacent voxels, from which 6 share a face, 12 share an edge and 8 voxels share only a corner in 3D space (Figure 1). Based on this, the adjacency relation N between two voxels is defined. Face-sharing voxels have adjacency 6, face-sharing and edge-sharing voxels have the adjacency of 18, and 26-adjacent voxels are those that share a face, edge or corner. In 3D space N∈{6,18,26}, while in 2D N∈{4,8}. Accordingly, an N-path of voxels can be identified as a sequence of voxels, in which consecutive voxels are N-adjacent.

### 2.1. Common Voxelisation Properties

In contrast to vector representations, voxelised objects are prone to several configurations such as holes, cavities and penetration, which have to be taken care of during the voxelisation. The objects have to remain connected so that a discrete unit represents correctly the analogous representation of the vector object. This implies that the voxelisation algorithms should apply several topological constraints such as connectivity, separation, coverage and tunnelling [27].

**Figure 2:**
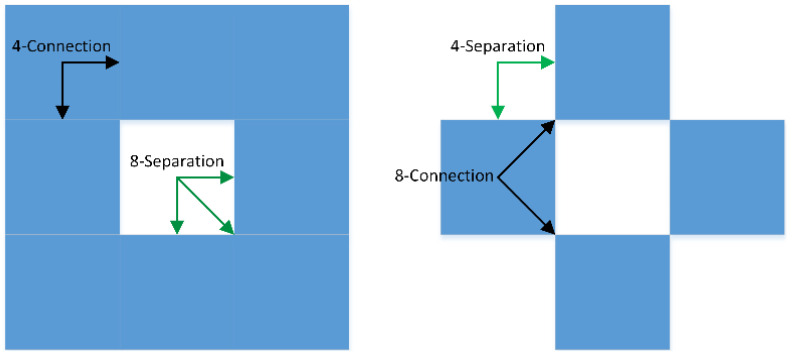
Connectivity and separation in 2D voxel set representing a circle. On the left, 4-connected voxelisation is achieved, which is at the same time 8-separating. On the right, 8-connected, and thus, 4-separating voxelisation is presented.

*Connectivity* identifies a set of *N*-paths between every pair of voxels belonging to an object. It indicates the result is an *N*-connected component, which is not disconnected in any way. Connectivity is a measure of the way voxels are linked to each other. It gives a notion of ‘thin’ or ‘thick’ voxelisation. Figure 2 illustrates ‘thick’ and ‘thin’ voxelisation, resulting, respectively, from 4-connectivity (connected via the edges) and 8-connectivity (connected via edges and vertices). The main benefits of strongly connected voxels, 8-connected in 2D and 18 and 26-connected in 3D are very attractive since they result in tinner objects with shorter length or area. In some cases, the connectivity of voxels might be insufficient to estimate the quality of the voxelisation process and therefore the notion of *separation* may need to be investigated.

*Separation* is a set of *N*-path voxels that divides two sets of voxels. This notion is intended to estimate how the “empty space” interacts with the voxelised object. The separation is exclusively a topological property, which does not reflect to what extent the actual object is correctly represented. The separation and connectivity are related. In 2D space, a 4-connecting voxelised object is always 8-separating and vice-versa (Figure 2). In 3D space, 6-connecting can be 18- or 26-separating and vice versa, but the relations are more elaborated. Separation is one of the main aspects that is considered in simulations. In the case of 6-separating voxelisation (i.e., thin voxelisation) a fluid would be allowed to go to voxels that only share a face with a voxel. In general, such a kind of voxelisation is sufficient since fluids could not travel to other voxels that share an edge or a corner with the central one. In favour of having 6-separating voxelisation is the fact that it is cheaper to compute and often more required in computer graphic applications [35]. The only reason when 18- or 26-separating voxelisation can be required is when an underlying model that computes the distribution of a gas or fluid takes into account values of diagonal voxels or allows distribution in those directions [36].

**Figure 3:**
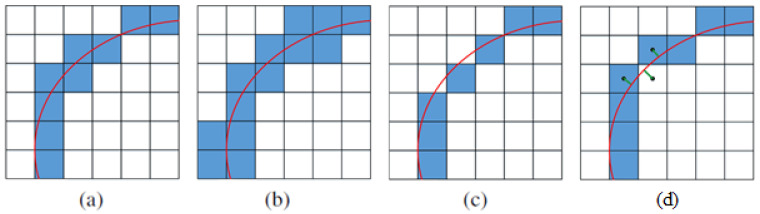
Representation of a cover (**a**), supercover (**b**), partial cover (**c**), and partial cover (**d**) well-voxelised curve in 2D.

*Coverage* is a notion that aims to define formally the thickness of a voxelised line or surface. There are three major variations of it: *cover*, *supercover* and *partial cover* (Figure 3). A set of voxels is called a cover if every point of an object is in a voxel. Normally, 8-connectivity in 2D creates a cover. A set of voxels are called *supercover* if all voxels that ‘contain’ or ‘touch’ points of the object are included in the set. A supercover can be obtained from algorithms that ensure 4- or 6-connectivity, respectively, 2D and 3D. A suvercover is also known as a *conservative voxelisation* [37,38]. It enlarges the object and may result in large overlapping parts of neighbouring objects [23]. However, for applications such as collision detection, occlusion culling and visibility processing conservative voxelisation is highly desirable [38]. In which case, voxel-based collision detection between two models would guarantee that those models do not intersect. A *partial cover* is a subset of cover, which allows for the maintenance of the tiniest voxelisation. As visible in Figure 3c this variation, while preserving well the shape of the object, may lead to cases when not all points of the object are voxelised. A partial cover, being not very restrictive, may lead to a connected voxel set that is not necessarily unique, as is visible in Figure 3c,d. Another approach solution can target a ‘well-voxelised’ approximation, which is tunnel-free and has a partial cover at the same time. To achieve such voxelisation, a method is proposed where the Euclidian distance is minimal between centroids of voxels representing a cover and the continuous object [27], resulting in keeping only them as part of the voxelised object (Figure 3d).

**Figure 4:**
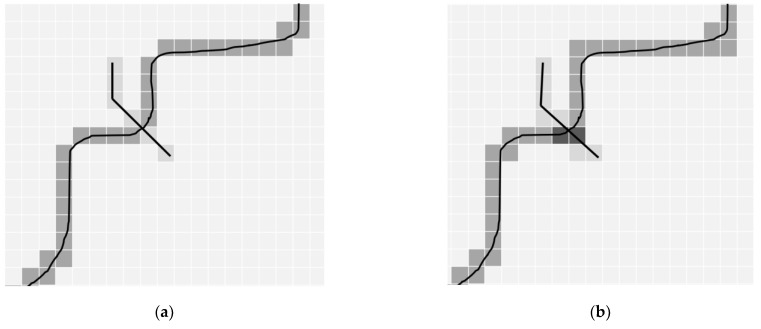
Intersection points of lines in 2D space: ‘tunnelling’, i.e., an intersection point is missing in 8-connected (**a**) and two voxels (in dark) as an intersection point (**b**).

Connectivity, separation and coverage allow to control the ‘thickness’ of voxelisation and therefore have the ability to indicate intersections and detect penetrations. For example, in a vector space, two lines intersect in a well-defined intersection point and a line cannot penetrate a closed polygon. However, depending on the voxelisation approach, the result might be different in the voxel space. For example, the intersection point of two lines in 2D can disappear if 8-connecting voxelisation is applied. (Figure 4, left). Alternatively, the intersection point might become enlarged and therefore fuzzy, if a 4-connecting approach is applied. (Figure 4, right).

*Tunnelling* is the notion to indicate the effect of penetration of two voxelised lines or surfaces (Figure 4, left). The 8-connected voxelisation in 2D, and 18- and 26-connected in 3D are prone to tunnelling. Consequently, a 4-connected voxel path in 2D and 6-connected in 3D are tunnel-free. In computer graphics, tunnelling may give the impression of having holes during rendering [23]. In applications performing analysis on voxelised objects, tunnelling might lead to difficulties in detecting intersections and penetrations.

### 2.2. Binary and Non-Binary Voxelisation

A common voxelisation classification depending on the resulting output identifies two types of voxelisation: binary and non-binary. Binary voxelisation is investigated by many researchers [3,39,40,41], but we need to explain the needs and possibilities of using non-binary voxelisation.

The main advantages of using binary voxelisation over a non-binary one is memory requirements, where only a single bit is needed to indicate a voxel’s status, and speed to create a voxelised object. Apart from a discrete representation of an object that binary representation provides, many times storing additional information is needed, especially for objects that come with textures, semantics and other properties. For example, we can store information related to surface normal and material properties like colour, opacity, density, depth, etc. There are several classes of non-binary voxelisation targeting anti-aliasing [29,42], multi values [43,44,45,46], and distance transform [47,48,49,50].

For example, to achieve alias-free rendering, estimating surface normal at the voxel’s location is required. To ensure correct voxel estimation the use of filters during voxelisation and reconstruction is needed [51]. As mentioned above, in addition to occupancy in multivalued voxelisation other information can be stored such as colour, material, opacity, etc. [45]. However, two nearly coplanar close surfaces representing two objects can fall in the same voxel, where storing information of both objects in a voxel is suggested [46]. Therefore, thin objects such as clothes or canvas placed close to other objects depending on an observer side could require keeping information of both objects in a voxel.

A distance map or field keeps distance at each point to the closest point of an object in space. Distance maps represent useful information for many spatial analyses and fluid simulations. Once a distance transform is signed, we can determine if a point is within or out of an object [49]. The use of distance transform is versatile including computer graphics, digital image processing (e.g., blurring effects, skeletonizing), path planning and pathfinding [20].

## 3. Voxelisation of 3D Geometric Primitives

As shown in the previous section, during the voxelisation of 3D objects we can deal with many aspects. A large number of voxelisation approaches have been reported in the literature, for example: for lines [23], triangles [24], polygons [25], parametric surfaces [42], implicit surfaces [52], constructive solid geometry [53], and polyhedral objects [54], etc. In this section, we will present the state-of-the-art methods for voxelisation of geometric primitives such as points, lines, triangles, surfaces and solids.

### 3.1. Point Voxelisation

Voxelisation of a point or many points can be done in a very straightforward way and be performed in several steps: (1) a translation according to a pivot point obtained from the bounding box of the points, (2) division of all coordinates considering a voxel size, (3) rounding the final values down to the first integer value, which can be a corner or centre point of the voxel and (4) recording a voxel in the voxel space if not existent. An algorithm considering the integer values for centroids of voxels is presented in [55]. Due to its simplicity to calculate and the ability to run in parallel, a point voxelisation technique known as particle-in-cell (PIC) is used in physical simulations to track the movement of densities and currents in voxel space [56].

### 3.2. Line Voxelisation

Going through the voxelisation process, a 3D continuous line should be transformed into a discrete set of connected voxels. A discrete line representation can have many purposes and roles [23]. A voxelised line or line segment is fundamental primitives which can be used as a building block for generating more complex 3D objects. For example, if we extend a circle following a line direction we can ending up with an open cylinder. Another example of using line voxelisation techniques is for ray traversal in voxel space. In this case, a set of voxels intersected or visited by the continuous ray can be determined. Line voxelisation algorithms can be also used in voxelisation of triangles [41].

In 3D, 6-connected and 26-connected line voxelisation techniques are usually discussed by researchers. The main advantage of 26-connected line voxelisation is the lower computational cost since it generates approximately two times fewer voxels [23]. Another type of line voxelisation that needs to be discussed is spline voxelisation since not all lines are straight.

#### 3.2.1. 6-Connected Voxelisation Algorithms

A straightforward method of raymarching voxels in a uniform grid was proposed generating a 6-connected path [1]. This method is nowadays known as the real line voxelisation (RLV). The method traverses the intersections between a line and the grid. The next intersection is identified based on the proximity along the axes and line direction. Thus, the method identifies during each step the intersecting points with the grid in all three dimensions and shifts the focus to the closest point for the next iteration. If the line passes through the corner of the voxel grid, an arbitrary voxel candidate can be picked or supercover line voxelisation (SLV) can be formed by labelling all touching voxels as filled.

Another algorithm worth mentioning producing 6-connected voxelisation is Xiaolin Wu’s line algorithm [57]. This algorithm is commonly used in modern computer graphics because it supports antialiasing while being fast compared to other available algorithms.

**Figure 5:**
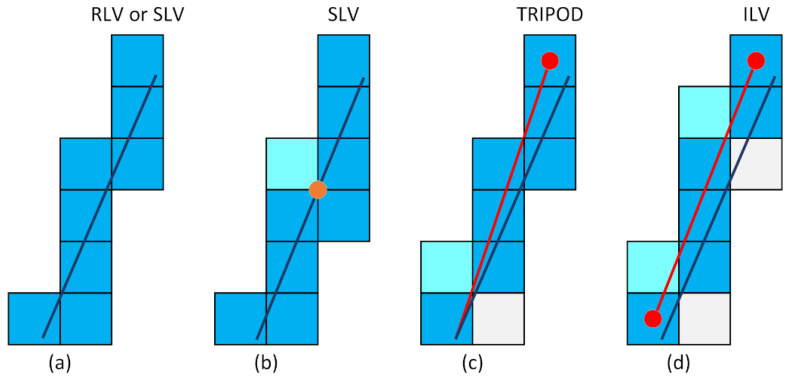
(**a**) RLV or SLV; (**b**) SLV generates more voxels than RLV considering all touching voxels; (**c**,**d**) small variations of the voxels coverage generated by Tripod and ILV algorithm.

A method generating a 6-connected line, named tripod, is proposed suggesting a comparable performance in voxelisation speed. The method is tracking the projections of a line on the three main axes [23]. Although this method requires the line of origin to lay at the centre of the voxel to avoid fractions in calculations, it suggests having the containment property. However, this might not be the case since every shift can result in some other voxels being covered (Figure 5c).

Following the same structure as RLV, a new approach was presented called integer-only line voxelisation (ILV) [41]. The main idea of this method is to avoid floating-point arithmetic and divisions present in RLV. Apart from the shift of the starting point of a line as in the tripod algorithm, this algorithm shifts the endpoint to the voxel centre as well. This can cause covering of voxels that are not present in RLV, and thus not guaranteeing the containment property of the original line (Figure 5d).

#### 3.2.2. 26-Connected Voxelisation Algorithms

Many algorithms create thin line voxelisation. Regarding 8-connected rasterisation, the most famous algorithms are digital differential analyser (DDA) (https://en.wikipedia.org/wiki/Digital_differential_analyzer_(graphics_algorithm). (accessed on 30 November 2021)) and Bresenham’s line algorithm [58]. The main difference between these two algorithms is that Bresenham’s line algorithm employs integer with round off functions while the DDA algorithm works with floating-point values. Another pro of using Bresenham’s algorithm is the computational performance mainly due to using additions and subtractions compared to the DDA which uses multiplications and additions. Unlike the DDA algorithm, the Bresenham algorithm is an integer-only line voxelisation algorithm, requiring endpoints to lie exactly on the middle points of the grid. However, we should point out that this shift can result in the coverage of different voxels between these two algorithms. A 3D version of DDA [59] and Bresenham’s algorithm [60] are also proposed creating a 26-connected line, behaving in the same way as the initial algorithms.

#### 3.2.3. Spline Voxelisation Algorithms

**Figure 6:**
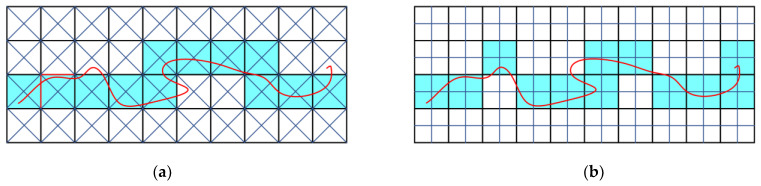
Voxelisation of a curve using intersecting targets in 2D. (**a**) Using cross-diagonal intersection targets forming 4-connected and 8-separating voxelisation. (**b**) Using crosshairs intersection targets forming 8-connected and 4-separating voxelisation.

To tackle a variety of lines, including parametric ones, Laine introduced two approaches considering intersections between specific targets and the grid [61]. Targets that are suggested are diagonal and crosshair ones. The targets can be applied in 2D and 3D voxelisations. Using one of these intersecting targets results in a voxelisation with different connectivity and separability properties. Generally, a diagonal target leads to 4-connectivity and a crosshair target to 8-connectivity voxelisation in 2D (Figure 6). The process is performed for each voxel which can be optimised by casting rays diagonally and horizontally from two directions for both scenarios in 2D or by intersecting planes in the same way for 3D. Another possible solution that we can think of is to approximate such a line with straight lines and use some of the previously mentioned algorithms to voxelise them.

#### 3.2.4. Comparison of Line Voxelisation Algorithms

There are numerous algorithms that can successfully perform line voxelisation in two or three dimensions. In Table 1, we present chronologically nine approaches and some of their characteristics. The main difference between the algorithms is the type of voxelisation that they achieve. They either use floating-point or integer arithmetic. When it comes to speed, integer-based algorithms achieving thinner voxelisation should be the quickest. However, this can depend on the application in which they are used. Thus, targeting optimal scanline voxelisation of 3D models RLV outperformed the 3D Bresenham’s line algorithm and ILV in terms of speed and accuracy of approximating original 3D models [62]. It is pointed out that the main reason for this is the consideration of many edge cases, in which case other algorithms were slower. Tripod and 3D-DDA were not considered in this study, although authors behind Tripod algorithm suggested that their algorithm can achieve comparable results to RLV [23]. However, we should mention that this is only one application area in which these algorithms can be compared.

### 3.3. Triangle Voxelisation

**Figure 7:**
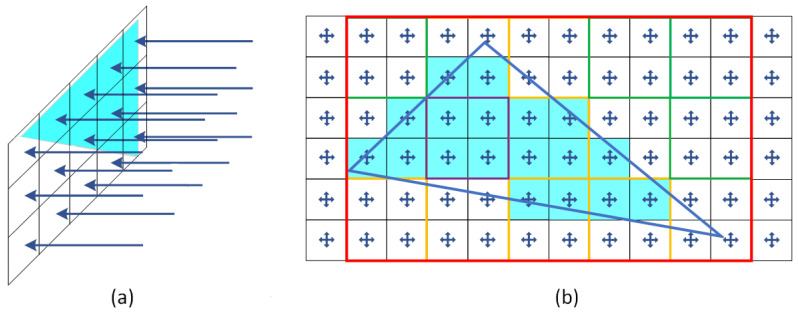
Triangle voxelisation. (**a**) uniform ray casting (**b**) rasterisation techniques; in red, the bounding box, in green tiles that are outside; in purple tiles that are inside; in yellow tiles that are intersecting the triangle are presented.

Triangles are the most basic polygons which have some unique properties such as being planar, having a well-defined interior and can perform quick intersections with rays. Triangles are almost always used as a building element of more complex objects like polygons and surfaces. In general, rasterisation is the main technique used for voxelisation of triangles, but they can be voxelised using ray casting as well (Figure 7). The first approach identifies which pixels to cover of a triangle in 2D space and reprojecting them into 3D space, while the second one relies on a quick intersection identification between rays and triangles.

#### 3.3.1. Rasterisation

Rasterisation approaches mainly rely on bringing triangles into 2D space to identify quickly which pixels should be filled using inside/outside checks. To optimise which pixels to check, as presented in Figure 7b, the simplest approach is to consider only pixels that are covered by the bounding box of a triangle [63]. However, for elongated triangles, many pixels that are outside still need testing, which can be considered as wasted computation. As a result, an approach tessellating a bounding box space into tiles is proposed (Figure 7b), which can quickly eliminate large blocks of pixels that are outside but also inside of a triangle [64]. At the same time, many rasterisation approaches target various pixel traversal ways to quickly identify which to fill. Thus, algorithms considering backtrack traversal [65], zigzag traversal [66], central-line traversal [63,67], tiled traversal [68,69,70] and midpoint traversal [71] are available. Tiled traversal algorithms are considered the best out of them, also reducing the needed power consumption. However, this might not be the case if triangles are smaller compared to a selected pixel size [72], requiring to split them into groups for faster processing [26].

DDA and Bresenham algorithms can be used for triangle rasterisation, but they can be difficult to implement in hardware resulting in a few approaches relying on them for triangle voxelisation [71]. However, researchers are trying to come up with new approaches that actually involve line rasterisation [41,62], achieving compareble preformance with above mentioned approaches.

#### 3.3.2. Raycasting

Many algorithms are targeting ray-triangle intersections to derive which triangles to render on a screen [73,74]. Ray casting can be performed from a specific point of view using a perspective camera or an orthogonal camera, where rays are uniformly sent towards an area of interest. Both approaches are commonly used in rendering scenes, keeping depth and other parameters (e.g., colour, reflectivity, etc.) for each pixel that is closest to a camera within Z-buffer. However, voxels do not cover the same area in the scene if a perspective camera is used. The latter approach considers casting rays from a uniform grid (Figure 7a) which can generate uniformed size voxels. Moreover, if all intersections with triangles are identified, the whole scene can be voxelised at once with the same size voxels. In order not to miss thin structures, raycasting can be performed in all three directions. To reduce the intersection checks between triangles and rays an algorithm is proposed which checks first if bounding boxes of triangles are inside the view frustum [75]. The same technique can be applied for any area of interest to eliminate quickly triangles that are not inside.

Similar techniques are used for the efficient calculation of ray-polygon intersections. For convex polygons dividing them into triangles and performing inside-outside check is suggested [76], while for non-convex and self-intersecting polygons odd/even parity can be used for a ray-polygon intersection [77]. Another set of algorithms achieving 6-connected and 26-connected voxelisation are presented [55], which are based on targets intersections previously mentioned in Section 3.2.3. These approaches can be considered as sending rays in all three directions and diagonally to achieve 26-connected and 6-connected voxelisation, respectively. These approaches are also extended to support surface voxelisation.

#### 3.3.3. Comparison of Triangle Voxelisation Algorithms

Rasterisation techniques are predominantly used for voxelisation of triangles (Table 2). Although ray casting techniques are slower than rasterisation ones, they can be used at the same time to obtain shadows and reflections more accurately for computer graphics applications. Using rasterisation it is possible to obtain different voxelisation properties, which are usually driven by application requirements. On the other hand, using raycasting it is not possible to achieve conservative voxelisation since edges of triangles can be easily missed. The rasterisation techniques rely on fast traversal of pixels within a bounding box or tiles, but it is possible to use line rasterisation techniques as well. Ray-triangle and ray-polygon intersection can be used to voxelise triangles and polygons, respectively.

### 3.4. Surface Voxelisation

A surface usually represents a continuous object resembling a deformed plane. There are many algorithms successfully performing surface voxelisation of 3D objects. To achieve a surface voxelisation there are two main approaches. The first approach is based on slicing of a scene or an object from one or more viewing directions. The second one considers rasterisation of triangles in 2D based on the dominant axis and the identification of overlapping voxels in 3D space. Another known classification divides approaches using graphics pipeline and computational voxelisation [78].

#### 3.4.1. Slice-Based

**Figure 8:**
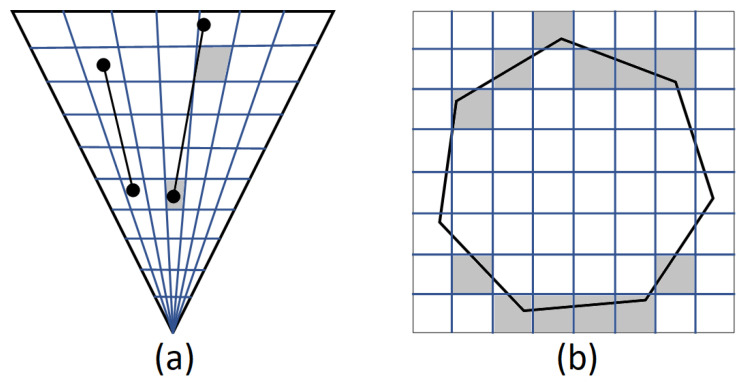
Limitations of one-side slicing seen from the bottom up. (**a**) view frustum of a perspective camera where the left object is completely missed, and the right object has disconnected voxels (**b**) depth slice of an orthogonal camera.

Regarding slice-based voxelisation, several algorithms are proposed [14,43]. The main idea behind these approaches is to perform voxelisation from a viewpoint slice-wise. The main disadvantages of these approaches are related to missing thin structures and having discontinuity between voxels (Figure 8), especially in the case of considering one viewing direction [3]. Thus, these approaches cannot guarantee that connectivity or any other property will be preserved during voxelisation. For instance, voxelising thin objects such as trees’ branches can easily have disconnected voxels. To capture all pixels overlapped by triangles and then identity for each pixel depth range along the viewing direction, conservative voxelisation is proposed [38]. This can definitely address the issues, but some additional voxels may be set in the depth range computation due to robustness problems [3].

#### 3.4.2. Rasterisation

Many approaches perform triangle rasterisation obtaining a voxelised model. Some of them use bounding boxes of triangles to test which voxels to cover [3,72,79], while others rely on a tile-based voxelisation [26,80,81] where triangles are assigned to each tile they overlap, which are checked sequentially for voxels coverage afterwards.

Using a triangle/box overlapping technique it is possible to assign one thread per triangle and test each pixel for coverage. This approach relies on a 2D axis-aligned box to test coverage. Tile-based approaches as discussed previously can boost voxelisation performance since not all pixels will be tested. Both approaches are suitable for running the voxelisation in parallel to achieve fast performance [3,26]. However, there can be a huge overhead if models are represented by small triangles compared to a selected pixel size [82]. To mitigate this issue Pantaleoni introduced coarse and fine rasterisation, where tiles are split based on the number of triangles during the coarse rasterisation for better load balancing in the fine rasterisation step afterwards. A similar approach was presented by Kalojanov [83] concentrating more on fast rendering, keeping all overlapping triangles per voxel, where conservative voxelisation was not strictly identified. Another approach using point-tessellated voxelisation is proposed afterwards [40]. The method calculates a triangle tessellation factor to subdivide triangles into micro triangles, in which centroids are voxelised afterwards to obtain a voxelised model. However, this approach can miss voxelising some voxels and it is not necessarily quicker. Recent work uses line voxelisation at its core to identify filled voxels for each triangle of a surface model [41]. By using either SLV or ILV approach a suvercover or 26-tunnel-free surface voxelisation can be identified. The method can downgrade the voxelisation to generate tinner surfaces like 18- or 6-tunnel-free ones. A hybrid approach relying on tile-based rasterisation and raycasting is also proposed for effective rendering of surface and solid voxelisation [45].

#### 3.4.3. Comparison of Surface Voxelisation Algorithms

Table 3 shows the most recent methods used to achieve fast surface voxelisation, where most of them use rasterisation to identify a voxel representation. Some of the methods are more flexible and can achieve with small modifications different voxelisation properties, whereas others are more specific. Researchers used a variety of techniques to acquire surface voxelisation for different applications. It is hard to tell which method is the most robust, but possibly the methods by Pantaleoni [26] and Zhang [41] can be suggested as the best. In comparison, the method presented by Zhang outperformed the ones presented by Pantaleoni, where the ILV method generated slightly more voxels. However, it is pointed out by the authors that additional information (colour, surface normal vectors, and so on) cannot be stored during voxelisation, which is not the case with Pantaleoni’s approaches.

### 3.5. Solid Voxelisation

As opposed to voxels covering a shell (i.e., surface) of an object, in a solid voxelisation, voxels whose centroids are inside the object are taken into account. Sometimes a boundary representing the surface of a solid object can be voxelised as well [43]. Methods utilised to acquire solid voxelisation are similar to the surface ones, considering either rasterisation [3] or slicing [2,28,39,43,84]. Achieving quick solid voxelisation is a less studied topic than surface voxelisation mainly due to its need and speed to acquire interior voxels of a 3D model. However, solid voxelisation can be used for translucency effects, volume visualisations used to show CT scans, particle collision detection and interaction, morphological operations, and CSG operations [39]. By counting the number of voxels representing an object, volume can be calculated [85], which can be identified even more accurately if object-voxel coverage factor is recorded [3].

**Figure 9:**
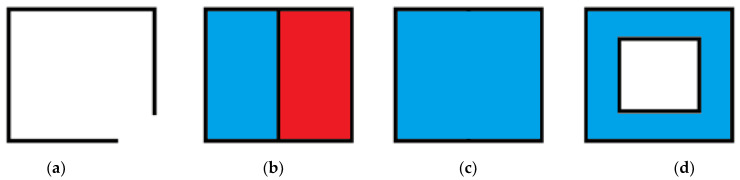
Different 3D solids. None-watertight models: enclosed object (**a**), object with an inner wall (**b**). Watertight solid models: enclosed object (**c**), object with a hole (**d**).

However, all current approaches are concentrating on solid voxelisation of watertight models. In watertight models, all points of each connected component have a clear separation between interior and exterior. For example, a point in space belongs to an interior or exterior if the number of intersections of a ray with the model from that point in any direction is odd or even, respectively (Jordan theorem) (https://en.wikipedia.org/wiki/Jordan_curve_theorem (accessed on 30 November 2021)) [86]. Figure 9 shows the difference between watertight and non-watertight models.

#### 3.5.1. Slice-Based

A slice-based algorithm for solid voxelisation using a clipping plane to generate a 2D slice is proposed [43], where a logical XOR operation between the previous and current slices are used to achieve a solid voxelisation. However, only a binary voxelisation is generated due to XOR operations, and some voxels can be missed. An approach relying on surface voxelisation and consequent 2D scan-filling in all three directions is proposed [84]. This approach suggests encoding binary voxels in separate bits of multiple targets, enabling processing many slices in a single pass. However, the algorithm fails if two fragments are located in the same voxel. An algorithm achieving multi-valued solid voxelisation is suggested using depth buffer and stencil buffer to create a mask for solid slice creation [28]. Building on top of their previous research [2], an approach for solid voxelisation is presented where all slices are processed at a time using more robust bitwise OR operation [39]. The authors also presented how to achieve solid conservative voxelisation, combining their solid voxelisation and conservative surface voxelisation of Zhang [38].

#### 3.5.2. Rasterisation

Two types of triangles-based solid voxelisation are presented [3]. The first one is based on rasterisation of each triangle considering their bounding boxes, while the other one assigns triangles into tiles to speed up the voxelisation process for situations when the grid size is high, or the model contains many triangles. For both approaches, flipping the voxels in one direction is necessary to achieve solid voxelisation. The authors proposed one more approach which uses sparse octree performing first slightly modified conservative surface voxelisation to identify active voxels that will be stored in an octree, followed by hierarchical inside/outside propagation to achieve solid voxelisation. This approach is slower than the other two, whereas it requires less memory and enables direct rendering into a sparse spatial data structure.

#### 3.5.3. Comparison of Solid Voxelisation Algorithms

Table 4 shows the most recent approaches used for solid voxelisation, where the approaches by Schwarz and Seidel [3] and Eisemann and Décoret [39] represent state-of-the-art methods for solid voxelisation. In general, Schwarz and Seidel approaches outperformed the approach of Eisemann and Décoret for smaller grid sizes, while the former one was faster for more complex models. The inner part of objects is usually voxelised, but these methods can be relatively easily extended to obtain the surface’s voxels at the same time.

## 4. Voxel Data Technology and Structures

To quickly perform some computational tasks related to voxels and voxelisation the use of a graphics processing unit (GPU) is suggested. Apart from using GPUs to perform calculations and render some output, they can be used purely for fast computation, where the results are stored in video memory as data. At the same time, there are different ways to store the output while performing a voxelisation, which can be immediately optimised for usage in different application domains.

### 4.1. Voxel Hardware Technology

Fixed-functions and programmable pipelines are two techniques used to hardware-accelerate 3D data processing. In the last decade, programmable pipelines are mainly used due to their flexibility to configure not only the rendering pipeline but also the way vertices are transformed and lighting is calculated. To do any type of computations by a GPU, an API is needed to interact with it. For computational purposes, the most common APIs include CUDA (https://developer.nvidia.com/cuda-zone (accessed on 30 August 2021)) and OpenCL.(https://www.khronos.org/opencl/ (accessed on 30 August 2021)) In terms of rendering, APIs like OpenGL (https://en.wikipedia.org/wiki/OpenGL (accessed on 30 August 2021)), DirectX (https://en.wikipedia.org/wiki/DirectX (accessed on 30 August 2021)) and Vulkan (https://www.vulkan.org/ (accessed on 30 August 2021)) are commonly used, which can perform calculations but this is not their general purpose [62].

Many voxelisation approaches have taken the advantage of a GPU to perform fast voxelisations [2,3,26,28,35,39,45,87,88]. Additionally, the use of general purpose graphics processing units (GPGPUs) has become a common tool for high-performance computing, which allows access to many GPUs and parallelisation of many computational tasks [82].

### 4.2. Voxel Data Structures

Considering the structure of discrete objects, the results can be structured as a regular grid [89], general 2D lattices [90], distance transform [47], sparse octree [3,35], inverse sparse octree [91], sparse block grid [30], dynamic tubular grid [32], volumetric dynamic grid [33], sparse paged grid [34], etc.

As we can see, there are many voxel data structures, where each can be evaluated separately. For simplicity, we split the methods into static and dynamic grids based on the frequency to update the data structure. This does not indicate that methods evaluated as part of static grids cannot change or recreate the data structure, but it is not their general purpose. For example, rendering as an application area can be considered requiring static grids, while simulations performing constant updates need a dynamic grid data structure.

#### 4.2.1. Static Grids

The use of a regular grid is the most standard way to represent voxelised objects. Voxels are stored as three-dimensional arrays, which allows for quick and easy check of neighbouring voxels. Storage and fast retrieval of voxels to perform different analyses and tasks are also investigated suggesting the use of a multidimensional array database system which is called RasDaMan (https://rasdaman.com/ (accessed on 30 August 2021)) [92]. The approach relies on SQL-based arrays using a flexible tiling system and compression. However, to represent large objects or scenes a more space-efficient representation is usually needed. This transformation can be performed after a voxelisation, while some approaches are even suggesting a direct use of a hierarchical structure like sparse voxel octree (SVO) for surface [35] and solid voxelisation [3,70].

In this regard SVO is proposed as more memory efficiency keeping only the occupied voxels, fast culling and collision tests using ray casting, and adjustable depth level which implicitly provides a levels-of-detail mechanism [93,94]. However, SVOs only allow handling moderate scene sizes and resolutions while requiring relatively high memory cost and memory bandwidth [95]. To go beyond the available memory an out-of-core approach is presented [96], being able to construct a sparse voxel octree from a triangle mesh. In this way, large scenes can be voxelised without exhausting a computer’s main memory.

For many extremely compact representations of high-resolution volumetric models, common in volume rendering, a compression method is presented [97], but the increased compression rates impact the decompression and traversal costs, making them hardly usable in other areas. To identify a more robust solution achieving a compact voxel representation with reasonable memory footprint and without decompression overhead, the use of a sparse voxel directed acyclic graph (SVDAG) is suggested [98]. The main idea behind this method is simply merging identical subtrees, creating a compact solution where nodes can share pointers to identical subtrees and still being as fast as regular SVOs and other octrees, since it does not affect the tracing process. For comparison, the authors suggest that with the use of an SVDAG it is possible to store 19 billion voxels representing 128 K^3^ scene size, a required memory on a GPU is 945MB, which is substantially less compared to an SVO requiring 5.1GB without even counting pointers. Considering this technique, a symmetrically-aware sparse voxel directed acyclic graph (SSVDAG) is presented suggesting memory compression of nearly two times as opposed to SVDAG [95,99]. This method in addition to the original SVDAG method for nodes that are identified as similar creates tagged pointers on the level above which keep the transformation that needs to be applied to recover the original subtree, and compact similar nodes based on their occurrence frequency. By compressing arbitrary data such as colour, vectors normal and reflectance information apart from the geometry different methods are proposed [100,101,102].

Table 5 shows in the hierarchical order some of the most prominent static grid methods and their characteristics. One of the conclusions is that researchers in recent years are considering predominantly voxelised surface models which usually require a smaller memory footprint and even faster creation compared to solid voxelised models. We see the diverse use of technology to interact with voxel models, where CUDA, OpenGL and DirectX are mainly the GPU-based APIs used, which can indicate that they have high usability. When it comes to the voxel data structures, the main focus has been on minimising the memory output through some clever techniques allowing to render impressively large scenes. Attribute conservation was an important aspect to many researchers trying to deal with several characteristics at once required in computer graphics areas. Using out-of-core techniques has become a standard to process voxel data. The next steps are hard to predict, but we can definitely expect voxel data structures to efficiently manage city-scale models.

#### 4.2.2. Dynamic Grids

Data structures, to be efficient, are characterised usually by two attributes, memory efficiency and computational efficiency. Although it is relatively easy to design a fast (e.g., dense grid) or compact (e.g., octree) voxel data structure, it is very challenging to identify a data structure that possesses both. For example, tree-based approaches intend to reduce the needed memory footprint, but the main issue is slowness in accessing data and modification of data structure. This led to the development of many new sparse volumetric data structures to support simulations and real-time applications [105].

Sparse block grid (SBG) was introduced by Bridson [30] dividing a voxel model into smaller cubic blocks and keeping only pointers to occupied cubes which retains constant time access to grids. After that, the run-length encoding (RLE) method was introduced suggesting to compress regions from the narrow band while storing the narrowband regions with full precision [106]. To deal with both storage and computational requirements the dynamic tubular grid (DT-Grid) is identified [32]. This data structure is proposed as fast, cache efficient and low memory required. It can expand and contract freely without the need to predefine a bounding box. Hierarchical run-length encoded (H-RLE) grid [31] combines DT-Grid and RLE achieving slight improvements over DT-Grid [107].

Considering sparse time-varying volumetric data, a hierarchical voxel data structure is proposed [33]. It uses a Volumetric Dynamic grid that shares a few characteristics with B+trees (VDB), which considers spatial coherency of time-varying data to separately and compactly store data and grid topology. Thus, grid values can be stored out-of-core, keeping only grid topology in memory. The data structure is represented by a tree with a high branching factor having a large uniform grid at leaf nodes. OpenVDB (https://www.openvdb.org/ (accessed on 25 September 2021)) is an open-source version of this approach, which has been widely used due to its effectiveness. A sparse paged grid (SPGrid) data structure is proposed which allows storing simulation data in a pyramid of sparsely populated uniform grids optimising data access [34]. A GPU version of VDB is proposed [108], relying on dense n^3^ bricks to represent leaf nodes, where the grid size does not have to be predefined. This approach is extended afterwards with dynamic topology update for fluid simulations being able to deal with tens of millions of particles (https://github.com/NVIDIA/gvdb-voxels (accessed on 25 September 2021)) [109]. For physically and topologically complex material point method (MPM) simulations harnessing the power of GPUs and SPGrid, highly parallelised data structure is presented dealing with millions of particles [110]. To create a more robust method for high-performance computations on spatially sparse data structures Taichi language is proposed [111], which is open source (https://github.com/yuanming-hu/taichi (accessed on 25 September 2021)) as well. Apart from the high achievable performance, this language is greatly extendible and easy to learn to support different simulation demands. This programming language allows selecting between VDB, GSPGrid or even custom-based sparse data structure. Recently, NanoVDB data structure is introduced as a linearised version of an OpenVDB data structure [105], with several advantages including the use of GPUs, a stand-alone raytracer that is compatible with most graphics APIs, being fast and efficient in copying data between devices (e.g., CPU and GPU) and randomly accessing voxels, etc.

Table 6 shows the most prominent methods presented in the last two decades trying to come up with data structures dealing with efficient storage, fast random and sequential data access, calculations and rendering. Regarding the technology, GPU approaches are becoming dominant while also focusing on compatibility with more APIs. Researchers are identifying more advanced voxel data structures trading-off between several aspects. Most of the methods are relying on the available memory to perform all tasks, whereas OpenVDB and NanoVDB are using out-of-core methods which gives them the advantage to process unlimited size scenes, which is clearly observable based on the maximum tested grid size for OpenVDB. The general-purpose aspect shows that some of the data structures are problem-specific while others are more general. Simulations and rendering are the most commonly investigated aspects, where the support for additional functionalities like 3D deep learning increase the usability of a data structure.

## 5. Conclusions and Future Works

In this review, we see that voxelisation has been used in many areas, and it can be quite diverse in many aspects. In Section 2, we covered the main concepts considered in voxelisation such as connectivity, separability, coverage, as well as tunnel-freeness. We can say that 26-connected voxelisation usually requires less time and memory to be created and stored compared to 6-connected and conservative voxelisation. A 6-separated voxelisation is usually sufficient for applications such as fluid simulations. Regarding coverage, it strongly depends on the area in which it is used. For instance, for collision detection, occlusion culling and visibility processing conservative voxelisation is highly desirable. Tunnelling can be addressed if 6-connected voxelised lines are used to send rays or to intersect with objects. The other option would be to consider either 6-connected or conservative voxelisation for all objects. The usage of binary and non-binary voxelisation is presented in Section 2.2, indicating that non-binary voxelisation might be even more applicable than binary one.

Regarding 3D primitives, we examined voxelisation of points, lines, triangles, surfaces, and solids. In terms of line voxelisation, we split the algorithms into three groups, where the first two deal with different types of connectivity, while the third group focuses on voxelisation of curves. In terms of triangles, we examined how using rasterisation and raycasting triangles can be voxelised. For surfaces and solids, we identified that slice-based and rasterisation techniques are mainly used to perform voxelisation. We can see that surface voxelisation is investigated by the greatest number of researchers. There are several reasons for this. Firstly, surface voxelisation achieves smaller memory output and is faster compared to solid voxelisation. Secondly, some of the very common requirements such as collision detection, fluid simulations and ray tracing can be easily achieved over them. We should point out that all algorithms for solid voxelisation concentrate on the voxelisation of watertight models, which is not necessarily sufficient for some application domains like building information modelling (BIM) where objects may have overlaps.

In Section 4, the current technology used to perform voxelisation and to deal with voxels in general is elaborated. It is pointed out that all the newest approaches rely on a fast performance achieved using GPUs. We saw that there are many voxel data structures identified so far. We divided the data structures into static and dynamic grids considering the frequency to update a data structure. Real-time storage of voxels using SVO is suggested for larger objects reducing the memory requirements and allowing quick ray tracing. The memory footprint can be reduced even further if SVDAG and SSVDAG are used as data structures. Regarding dynamic grids, the state-of-the-art voxel data structure is NanoVDB which is superior to the rest in terms of speed as well as support for out-of-core processing which can handle infinite size scenes and models.

We presented here a vast number of algorithms targeting voxelisation of different geometric primitives. However, the greatest challenge is the availability of these algorithms for testing and further research, which usually requires building them from scratch in a possibly nonoptimal way. Therefore, creating a library with these algorithms would definitely facilitate the development of new approaches for voxelisations. Another even more interesting idea is to develop a database that would also use some of the presented data structures to effectively store and process voxels. For instance, RasDaMan uses arrays and tiles to manipulate voxels, which can be greatly improved using more advanced voxel data structures. At the same time, many algorithms currently used in image processing can be speeded up with the use of these voxel data structures. This will allow understanding in which scenarios the presented voxel data structures perform better compared to the rest.

## Figures and Tables

**Table 1:** Line voxelisation algorithms.

Method	Type	Property	General Purpose
2D Bresenham’s line algorithm [58]	Integer-only	8-connected	Line primitives rasterisation
2D-DDA	Floating-point or integer	8-connected	Line primitives rasterisation
3D-DDA [59]	Floating-point or integer	26-connected	Line primitives voxelisation
RLV & SLV [1]	Floating-point	Conservative	Line primitives voxelisation
Xiaolin Wu’s line algorithm [57]	Floating-point	Conservative	Antialiasing
Tripod [23]	Integer	6-connected	Line primitives voxelisation
3D Bresenham’s line algorithm [60]	Integer-only	26-connected	Line primitives voxelisation
Targets-based approaches [61]	Floating-point	6/26-connected	Irregular line primitives voxelisation
ILV [41]	Integer-only	6-connected	Surface voxelisation

**Table 2:** Triangle voxelisation algorithms.

Method	Type	Property	Main Technique
[3,63,65,66,67,71]	Rasterisation	6/26-connected & conservative	Bounding box, backtrack, zigzag, central-line, and midpoint traversal
[3,26,68,69,70]	Rasterisation	6/26-connected & conservative	Tile-based
[55,73,74,75,77]	Raycasting	6/26-connected	Ray-triangle and ray-polygon intersection
[41,62]	Rasterisation	6/26-connected & conservative	Line rasterisation

**Table 3:** Surface voxelisation algorithms.

Method	Type	Property	Main Technique	General Purpose
[43]	Slice-based	‘26-connected’	Plane slicing	Rendering
[14]	Slice-based	‘26-connected’	Depth peeling	Rendering
[38]	Slice-based	Conservative	Bounding box	Collision detection
[72]	Rasterisation	26-connected	Bounding box	Rendering
[79]	Rasterisation	26-connected	Bounding box	Rendering
[81]	Rasterisation	26-connected	Tile-based	Voxelisation
[3]	Rasterisation	Conservative & 26-connected	Bounding box	Voxelisation
[83]	Rasterisation	‘Conservative’	Two level grids	Rendering
[26]	Rasterisation	Conservative & 26-connected	Tile-based & bucketing	Voxelisation & rendering
[40]	Rasterisation	26-connected	Point tessellation	Voxelisation
[55]	Raycasting	6/26-connected	Intersecting targets	Voxelisation
[41]	Rasterisation	6/26-connected	ILV	Voxelisation
[45]	Rasterisation & raycasting	Conservative	Tile-based + ray-triangle intersection	Voxelisation & rendering

**Table 4:** Solid voxelisation algorithms.

Method	Type	Property	Main Technique	General Purpose
[43]	Slice-based	Interior only	Plane slicing	Voxelisation
[84]	Slice-based	Interior only	Surface voxelisation + 2D scan-filling	Voxelisation
[2]	Slice-based	Interior only	Bitwise OR operation	Rendering
[28]	Slice-based	Interior only	Mask creation	Voxelisation
[39]	Slice-based	Interior only & conservative	Single pass & bitwise OR operation	Voxelisation
[3]	Rasterisation	Interior only	Tile-based, bounding box, sparse octree	Voxelisation & storage

**Table 5:** Static grids methods.

Method	Geometry Voxelisation Type	GPU API/CPU	Voxel Data Structure	Attribute Conservation	Out-of-Core
[92]	Any	CPU	Regular grid	x	x
[39]	Solid	OpenGL & DirectX 10	Regular grid	x	-
[28]	Solid	OpenGL 2	Regular grid	x	-
[3]	Solid	CUDA	SVO	-	-
[93,94]	Surface	CUDA	SVO	x	-
[26]	Surface	CUDA	Regular grid	x	-
[40]	Surface	OpenGL 4 & DirectX 11	Regular grid	x	-
[103]	Surface	DirectX 11	SVO	x	-
[96]	Surface	CPU	SVO	-	x
[98]	Surface	CUDA	SVDAG	-	x
[104]	Surface	CUDA	SVO	x	x
[95,99]	Surface	OpenGL	SSVDAG	-	x
[100]	Surface	GPU	SVDAG	x	x
[101,102]	Surface	CUDA	SVDAG	x	x

**Table 6:** Dynamic grids methods.

Method	GPU API/CPU	Voxel Data Structure	Out-of-Core	Maximum Tested Grid Size	General Purpose
[30]	CPU	SBG	-	2000 ^3^	Simulation and rendering
[106]	CPU	RLE	-	624 × 554 × 488	Rendering
[32]	CPU	DT-Grid	-	1024 ^3^	Fluid simulation
[31]	CPU	H-RLE	-	5K × 3K × 3K	Fluid simulation
[33]	CPU	VDB	x	15K × 900 × 500	Simulation and rendering
[34]	CPU	SPGrid	-	2K × 2K × 4K	Fluid simulation
[108]	CUDA	GVDB	-	2048 ^3^	Simulation and rendering
[109]	CUDA	GVDB	-	1056 × 288 × 768	Fluid simulation
[110]	CUDA	GSPGrid	-	512 ^3^	MPM simulation
[111]	CUDA, OpenGL, Apple Metal	GVDB, GSPGrid, custom	-	4096 ^3^	Simulation, rendering, and 3D deep learning
[105]	CUDA, OpenCL, OptiX OpenGL, DirectX12, WebGL, HLSL & GLSL	NanoVDB	x	/	Simulation and rendering

## Data Availability

Not applicable.
